# Neuroprotective Effect of Non-viral Gene Therapy Treatment Based on Tetanus Toxin C-fragment in a Severe Mouse Model of Spinal Muscular Atrophy

**DOI:** 10.3389/fnmol.2016.00076

**Published:** 2016-08-24

**Authors:** Sara Oliván, Ana C. Calvo, Amaya Rando, Mireia Herrando-Grabulosa, Raquel Manzano, Pilar Zaragoza, Eduardo F. Tizzano, Jose Aquilera, Rosario Osta

**Affiliations:** ^1^Laboratorio de Genética Bioquímica, Facultad de Veterinaria, Instituto Agroalimentario de Aragón (IA2), Centro de Investigación y Tecnología Agroalimentaria de Aragón, Instituto de Investigación Sanitaria Aragón, Universidad de ZaragozaZaragoza, Spain; ^2^Centro de Investigación Biomédica en Red en Bioingeniería, Biomateriales y Nanomedicina, Grupo AMB, Instituto de Investigación en Ingeniería de Aragón (I3A), Universidad de ZaragozaZaragoza, Spain; ^3^Centro de Investigación Biomédica en Red sobre Enfermedades Neurodegenerativas, Spain Institut de Neurociències and Departament de Bioquímica i de Biologia Molecular, Facultat de Medicina, Universitat Autònoma de BarcelonaCerdanyola del Vallès, Spain; ^4^Department of Physiology, Anatomy and Genetics, University of OxfordOxford, UK; ^5^Área de Genética Clínica y Molecular, Hospital Vall d’Hebron, Centros de Investigación Biomédica en RedBarcelona, Spain

**Keywords:** spinal muscular atrophy, *c*-terminal fragment of the tetanus toxin, muscle, spinal cord, autophagy, apoptosis, muscular atrophy

## Abstract

Spinal muscular atrophy (SMA) is a hereditary childhood disease that causes paralysis and progressive degeneration of skeletal muscles and spinal motor neurons. SMA is associated with reduced levels of full-length Survival of Motor Neuron (SMN) protein, due to mutations in the Survival of Motor Neuron 1 gene. Nowadays there are no effective therapies available to treat patients with SMA, so our aim was to test whether the non-toxic carboxy-terminal fragment of tetanus toxin heavy chain (TTC), which exhibits neurotrophic properties, might have a therapeutic role or benefit in SMA. In this manuscript, we have demonstrated that TTC enhance the SMN expression in motor neurons “*in vitro*” and evaluated the effect of intramuscular injection of TTC-encoding plasmid in the spinal cord and the skeletal muscle of SMNdelta7 mice. For this purpose, we studied the weight and the survival time, as well as, the survival and cell death pathways and muscular atrophy. Our results showed that TTC treatment reduced the expression of autophagy markers (*Becn1, Atg5, Lc3*, and *p62*) and pro-apoptotic genes such as *Bax* and *Casp3* in spinal cord. In skeletal muscle, TTC was able to downregulate the expression of the main marker of autophagy, *Lc3*, to wild-type levels and the expression of the apoptosis effector protein, *Casp3*. Regarding the genes related to muscular atrophy (*Ankrd1, Calm1, Col19a1, Fbox32, Mt2, Myod1, NogoA, Pax7, Rrad*, and *Sln*), TTC suggest a compensatory effect for muscle damage response, diminished oxidative stress and modulated calcium homeostasis. These preliminary findings suggest the need for further experiments to depth study the effect of TTC in SMA disease.

## Introduction

Motor neuron diseases, a group of heterogeneous neurological disorders such as Amyotrophic Lateral Sclerosis (ALS) and Spinal Muscular Atrophy (SMA), are characterized by varying degrees of motor neuron degeneration. In particular, SMA is an autosomal recessive motor neuron disease and the first genetic cause of infant mortality, characterized by the degeneration of motor neurons in the anterior horn of the spinal cord, resulting in muscular atrophy and weakness. SMA patients have a homozygous loss of the survival motor neuron 1 (*SMN1*) gene, but retain one or more copies of a nearly identical homolog, *SMN2*. Therefore this disease is the result of insufficient amounts of SMN protein and its levels are generally inversely correlated with the severity of the disease, hence making *SMN2* copy number the predominant modifier of the neuromuscular phenotype (Monani and De Vivo, 2014). In SMA pathology, the most striking component is the loss of alpha motor neurons in the ventral horn of the spinal cord, resulting in progressive paralysis and eventually premature death. Despite the time that has elapsed since the SMA genes were identified, the currently available treatments for patients affected by the disease are palliative, based on symptomatic treatments and supportive care (Monani and De Vivo, 2014). Nowadays, the most interesting therapeutic strategies are represented by molecular, gene and stem cell-mediated approaches which are focused on activating *SMN2* expression, modulating splicing of *SMN2* or replacing *SMN1* (Zanetta et al., 2014; d’Ydewalle and Sumner, 2015).

A promising therapeutic approach could be the non-viral gene therapy based on the use of atoxic *C*-terminal fragment of the tetanus toxin (TTC). Tetanus neurotoxin is a protein produced by *Clostridium tetani* that cause tetanus, a fatal condition characterized by painful and uncontrolled muscle contractions (Farrar et al., 2000). The toxin is synthesized as a single polypeptide and is posts-translationally modified to produce light and heavy chains linked by disulfide bonds (Turton et al., 2002). The catalytic domain of the toxin resides in the light chain, while the translocation and receptor-binding domains are present in the heavy chain (Montal, 2010). Moreover, the heavy chain consists of two non-toxic fragments, the *N*-terminal or translocation domain and the *C*-terminal or receptor-binding domain (Chen et al., 2012). The atoxic TTC heavy chain (hereafter called TTC) can be retrogradely transported to the central nervous system and may be linked to different molecules without apparent loss of biological activity. The ganglioside-binding properties of fragment C have demonstrated that the presence of polysialic acids within the gangliosides, such as GD1b (disialic acid residues attached to the internal galactose residue) and GT1b (disialic acid residues attached to the terminal galactose residue) in cell membranes are neccesary to enable the tetanus-toxin internalization and therefore the TTC internalization in neurons. Additionally, the neurotrophin receptor p75NTR and TrkB is also essential in the retrograde pathway of TTC, sharing with NGF and BDNF the same retrograde transport organelles (Calvo et al., 2012b). These features allow the use of TTC as a valuable biological carrier of therapeutic molecules such as reporter genes or neurotrophic factors to ameliorate the disease advances of neurodegenerative disorders (Toivonen et al., 2010). In ALS, the delivery of glial cell-derived neurotrophic factor (GDNF) or brain derived neurotrophic factor (BDNF) to the spinal cord is improved by conjugation with TTC after intramuscular administration (Ciriza et al., 2008; Calvo et al., 2011). In SMA, one therapeutic strategy could be based on increasing the levels of neuronal SMN, for this reason, the genetic fusion of SMN and TTC was applied to deliver exogenous SMN to the cytosolic compartment of motor neurons (Francis et al., 2004). Interestingly, *in vitro* and *in vivo* studies have also shown that TTC itself may well harbor neuroprotective properties (Toivonen et al., 2010; Calvo et al., 2012b). In this way, in the mouse model SOD1G93A of ALS, the intramuscular injection of naked DNA encoding for TTC ameliorated the decline of hind limb muscle innervation, significantly delayed the onset of symptoms and functional deficits, improved spinal motor neuron survival and prolonged lifespan (Moreno-Igoa et al., 2010).

In the light of these preliminary results, the aim of the present study was to evaluate the possible therapeutic effect of intramuscular delivery of a TTC-encoding plasmid in the spinal cord and the skeletal muscle tissues of an intermediate mouse model of SMA, the SMNdelta7 mouse. In order to evaluate the neuroprotective effects under TTC treatment, we firstly registered the weight and the survival time, and the survival rate to further analyze the expression of several genes related to neurodegenerative process, in particular, autophagy, apoptosis and muscle atrophy.

## Materials and Methods

The care and use of animals were performed accordingly with the Spanish Policy for Animal Protection RD53/2013, which meets the European Union Directive 2010/63/UE on the protection of animals used for experimental and other scientific purposes. The in-house Ethic Committees for Animal Experiments of the Universidad de Zaragoza and Universitat Autònoma de Barcelona approved all of the experimental procedures.

### Spinal Cord Organotypic Culture and TTC Supplementation

Spinal cord organotypic cultures were obtained from lumbar spinal cords of 8-day-old Sprague-Dawley rat pups as previously described (Herrando-Grabulosa et al., 2013). Cultures were let to stabilize for 1 week, and after this point the medium was changed twice per week until 15 days *in vitro* (DIV).

TTC protein used in spinal cord organotypic culture treatments were purificated as previously described (Herrando-Grabulosa et al., 2013). TTC protein was added to the warm-fresh culture medium at a concentration of 10 nM.

### Immunoblotting

Spinal cord slices were collected in lysis buffer, homogenated, and quantified by BCA assay. Equal amounts of protein (20 μg/well) were resolved in SDS-PAGE and transferred to nitrocellulose membrane. Membranes were blocked with 6% non-fat dry milk in phosphate buffered saline (PBS) for 1 h at room temperature (RT) and incubated overnight with the corresponding primary antibody diluted in blocking buffer (SMN, 1:500, BD Biosciences and anti-β-tubulin, Becton-Dickinson). After several washes, membranes were incubated for 1 h with an appropriate secondary antibody. Blots were developed using a chemoluminiscent mix and exposed to enzymatic chemoluminiscence (ECL) films (Amersham Pharmacia Biotech). Densitometry was carried out using ImageJ software.

### Immunofluorescence

For organotypic cultures, spinal cord slices were fixed with 4% paraformaldehyde (PFA) at RT for 1 h. Slices were then washed twice with PBS for 15 min, blocked with 5% normal horse serum and 0.2% Triton-X-100 in PBS, and incubated overnight at 4°C with antibody against mouse anti-neurofilament heavy-chain (NF-H) (SMI-32; 1:1000, Sternberger Monoclonals Inc.) and rabbit anti-survival motor neuron (SMN; 1:50, Santa Cruz). Slices were then thoroughly washed in PBS with 0.2% Tween-20 and incubated with appropriate secondary antibody Alexa Fluor^®^555 goat anti-rabbit IgG (1:500) and Alexa Fluor^®^488 goat anti-mouse IgG (1: 1000) diluted in blocking buffer for 1 h at RT. Then, slices were incubated for 20 min with 4′-6-Diamidino-2-phenylindole (DAPI). Finally, slices were mounted in Superfrost^®^Plus slides (Thermo Fisher Scientific) with Fluoromount-G mounting medium (SouthernBiotech) and fluorescence was visualized under epifluorescence microscope (Nikon Eclipse 90i; Nikon Instruments Inc.) or Olympus FluoViewTM FV1000). Motor neurons in the organotypic spinal cord slices were identified by SMI-32 immunostaining on the basis of their morphology and size (>20 μm) and their localization in the ventral horn. A minimum of 15 sections were used for MN counting for each experimental condition.

In case of SMA mice, animals were perfused transcardially with cold 4% PFA in PBS. Spinal cords were dissected, post-fixed in PFA for 24 h, and transferred to 30% sucrose in PBS for at least 48 h at 4°C. Sample tissues were embedded in OCT (Tissue-Tek, Sakura Finetek) and frozen in liquid nitrogen-cooled 2-methylbutane. Transverse sections of spinal cord (10 μm) were cut with using a cryostat (CM1510S Leica Microsystems). Tissue sections were re-fixed on ice with formalin solution 10% (HT5014, Sigma) for 10 min, permeabilized with 0.1% Triton X-100 and 0.1% sodium citrate in PBS for 10 min at RT, and blocked with 10% goat serum and 1% BSA in PBS for 30 min at RT. After washes, sections were incubated with LC3 primary antibody (1:200; PD014, MBL) over night at 4°C, and subsequently incubated with Alexa Fluor 546 goat anti-rabbit IgG (Invitrogen) for 1 h at RT. Nuclear staining (in blue) was performed using a mounting medium with DAPI (Vectashield, H-1200, Vector Laboratories) and visualized on an Olympus IX81 fluorescence microscope.

### SMA Mice

Moderate Type II SMA mice FVB.Cg-Tg(SMN2^∗^delta7)4299Ahmb Tg(SMN2)89Ahmb *Smn1^tm1Msd^*/J were kindly provided by Dra. Lucía Tabares (Universidad de Sevilla). Transgenic Smn^+/-^;SMN2;SMNΔ7 mice were maintained as heterozygous breeding pairs in the Servicio General de Apoyo a la Investigación-SAI of the Universidad de Zaragoza.

This mouse model at birth is noticeably smaller than normal littermates. Signs of muscle weakness appear progressively after day 5 and the mouse displays an abnormal gait, shakiness in the hind limbs and a tendency to fall over with a lifespan of 13 days (Le et al., 2005). Body weight and survival measures were taken daily in the morning. To evaluate these parameters, we produced 9 l with 71 pups, 5 lwere injected with pCMV–TTC plasmid (TTC treatment) and 4 with pCMV plasmid (untreated). In relation of the 71 pups, 21 were SMA mice (10 treated with TTC and 11 untreated), 35 were heterozygous mice (25 treated with TTC and 10 untreated) and 15 were WT (7 treated with TTC and 8 untreated).

To study the gene expression by real-time PCR, we produced 9 l with 81 pups, 5 lwere injected with pCMV-TTC plasmid and 4 with pCMV plasmid. In relation of the 81 pups, 10 were SMA mice (5 treated with TTC and 5 untreated), 48 were heterozygous mice (25 treated with TTC and 23 untreated) and 23 were WT (14 treated with TTC and 9 untreated).

Finally, in relation to immunofluorescence assay, we produced 7 l with 43 pups, 5 l were injected with pCMV-TTC plasmid and 2 with pCMV plasmid. In relation of the 43 pups, 6 were SMA mice (3 treated with TTC and 3 untreated), 21 were heterozygous mice (12 treated with TTC and 9 untreated), and 23 were WT (10 treated with TTC and 6 untreated).

### Plasmid Purification and Intramuscular Injection

The TTC gene used in mice treatments was constructed in the pcDNA3.1 eukaryotic expression plasmid under the control of the cytomegalovirus (CMV) immediate-early promoter. pCMV:TTC was obtained by cloning a BamHI/NotI TTC fragment from the pGex:TTC vector (Coen et al., 1997) into pcDNA3.1 as previously described (Moreno-Igoa et al., 2010).

For the transformation assay, competent cells (*Escherichia coli* DH5α bacteria) were used, and the constructed plasmids were purified with QIAprep Spin Miniprep kit (QIAGEN). The sequence of the purified plasmids (BigDye Terminator v3.1 Cycle Sequencing kit, Applied Biosystems) was checked to confirm that the cloned DNA fragments were correctly inserted in the vectors. The recombinant plasmids were finally expanded in DH5α bacteria and purified using the EndoFree Plasmid Mega Kit (Qiagen). The recombinant plasmids were subjected to 1% agarose gel in 1X Tris-boric-EDTA (TBE) to yield fragments of the expected molecular weight. The quantity of the obtained recombinant plasmids was measured using a NanoDrop^®^ Spectrophotometer (ND-1000 V3.3.0).

At post-natal day 1 (P1) mice were immobilized via cryoanesthesia and injected intramuscularly with 100 μg of naked DNA encoding for pCMV-TTC or non-coding pCMV plasmids into the *quadriceps femoris* muscles (one injection with 50 μg per muscle).

At P7 (early-symptomatic), the pups were lightly anesthetized with isoflurane and were euthanized by rapid decapitation. The skeletal muscle and spinal cord tissues were harvested, snap-frozen in liquid nitrogen, and then stored at -80°C for RNA extraction.

### Quantitative PCR

Spinal cord and muscle tissue were pulverized in liquid nitrogen with a cell crusher. For RNA extraction, powdered samples were resuspended with Trizol Reagent (Invitrogen). RNA extracted was treated to eliminate genomic DNA using the Turbo DNA-freeTM kit (Ambion) and the reverse transcription was carried out according to the SuperScript^TM^ First-Strand Synthesis System kit (Invitrogen). Gene expression was assayed by real-time PCR in a StepOne^TM^ Real-Time PCR System (Applied Biosystems). Primer and probe mixtures for each gene of interest were supplied by Applied Biosystems (**Table 1**). Two endogenous genes (*Gapdh* and *β-actin*) were used for normalization of the data. All reactions were performed in triplicate and all reaction efficiencies of the primer/probe sets were close to 100%. Target gene expression was normalized using the geometric mean of these two housekeeping genes and relative gene expression was determined using the 2^-ΔΔCT^ method.

**Table 1:** Taqman^®^ probe and primer mixtures used in gene expression assays.

Name	Gen symbol	Part number
Autophagy related 5	*Atg5*	Mm00504340_m1
Beclin 1, autophagy related	*Becn1*	Mm00517174_m1
E2F transcription factor 1	*E2f1*	Mm00432939_m1
Microtubule-associated protein 1 light chain 3 alpha	*Map1lc3a (Lc3)*	Mm00458724_m1
Sequestosome 1	*Sqstm1 (p62)*	Mm00448091_m1
BCL2-associated X protein	*Bax*	Mm00432050_m1
B cell leukemia/lymphoma 2	*Bcl2*	Mm00477631_m1
Caspase 1	*Casp1*	Mm00438023_m1
Caspase 3	*Casp3*	Mm01195085_m1
Ankyrin repeat domain 1	*Ankrd1*	Mm00496512_m1
Calmodulin 1	*Calm*	Mm00486655_m1
Collagen type XIX alpha 1	*Col19a1*	Mm00483576_m1
F-box only protein 32	*Fbxo32*	Mm01207878_m1
Metallothionein 2	*Mt2*	Mm00809556_s1
Myogenic differentiation 1	*Myod1*	Mm00440387_m1
Paired box gene 7	*Pax7*	Mm00834079_m1
Ras-related associated with diabetes	*Rrad*	Mm00451053_m1
Sarcolipin	*Sln*	Mm00481536_m1
Survival motor neuron 1 (SMN 1)	*Smn1*	Hs00165806_m1
Glyceraldehyde-3-phospate dehydrogenase	*Gapdh*	4352932E
Actin, beta, cytoplasmic	*Actb (β-actin)*	4352933E

*Gapdh and *Actb* (β-actin) were used as housekeeping genes.*

### Statistical Analysis

Statistical significance was determined by one-way ANOVA followed by Bonferroni’s *post-hoc* test. Survival data was analyzed using the Kaplan-Meir test. All of the values were expressed as means and error bars represent standard error of the mean (mean ± SEM). The statistical significance threshold was set at *p* < 0.05.

## Results

### TTC Treatment Increase SMN Expression in Motor Neurons

**FIGURE 1:**
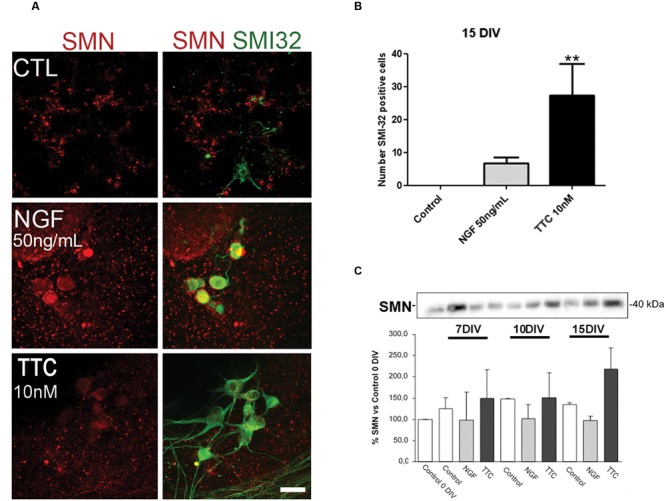
**Enhanced levels of SMN protein after TTC treatment. (A)** Representative images of the ventral horn hemisections immunolabeled against SMN protein (red) and motor neuron marker SMI32 (green). Scale bar: 50 μm. **(B)** Bar graph showing the number of SMI-32 positive cells at the ventral horns with a diameter >25 μm at 15DIV alone or under the treatment of NGF (50 ng/mL) or TTC (10 nM). Values are the mean ± SEM of at least 15 sections per treatment. ^∗∗^*p* < 0.01. **(C)** Evaluation by western blot analysis the levels of SMN protein under the treatment of NGF 50 ng/mL and TTC 10 nM along the progression of the spinal cord organotypic culture (0, 7, 10, and 15DIV). All results were from at least two independent experiments. Equal amount of protein was added to each well.

Prior to the *in vivo* assays, the effects of TTC on SMN expression and motor neuron survival were studied *in vitro*. In spinal cord organotypic cultures, TTC protein enhanced levels of SMN and also significantly increased the number of motor neurons (**Figures 1A,B**). This effect was more evident than the observed in the case of NGF supplementation. These results suggested that TTC achieve a neuroprotective effect in motor neurons. Moreover, immunoblotting quantification of SMN showed that its expression was increased along the days of culture and reached the highest level at 15DIV (**Figure 1C**).

The next step and prior to performing the assay in our severe mice model of SMA, the non-viral gene therapy with pCMV-TTC was assessed *in vivo* in a mouse model of motor neuron disease (Supplementary Material S1). The results obtained showed that, 10 days after inoculation, TTC treatment significantly increased the levels of the SMN gene in muscle and spinal cord tissues in this animal model.

### Improvement of the Autophagy and Apoptosis Markers Under TTC Treatment

**FIGURE 2:**
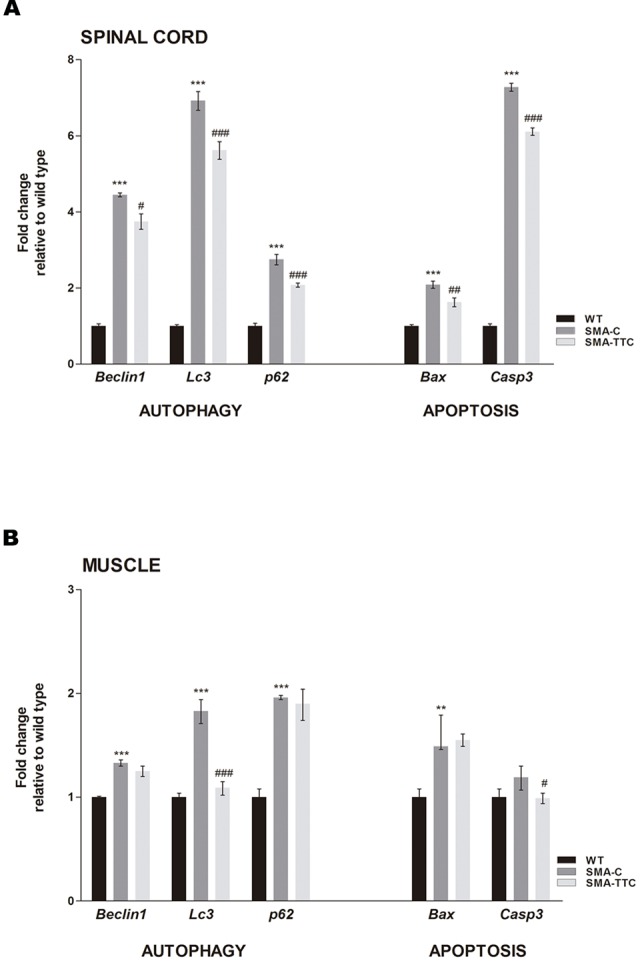
**Effect of TTC-encoding plasmid in autophagy and apoptotic markers.** Relative expression values of autophagy (*Becn1, Lc3*, and *p62)* and apoptotic (*Bax* and *Casp3*) genes in WT mice (WT, black bars), untreated SMA mice (SMA-C, dark gray bars) and SMA mice treated with TTC (SMA-TTC, light gray bars) in spinal cord **(A)** and skeletal muscle **(B)**. Each data point represented the mean ± SEM, *n* = 5 animals per group. WT *versus* SMA-C: ^∗∗^*p* < 0.01 and ^∗∗∗^*p* < 0.001. SMA-C *versus* SMA-TTC: ^#^*p* < 0.05, ^##^*p* < 0.01 and ^####^*p* < 0.001.

mRNA expression levels of the well-known markers of autophagy (*Becn1, Lc3*, and *p62*) and apoptosis (*Bax* and *Casp3*) were quantified by real-time PCR in spinal cord and skeletal muscle samples of WT and SMA mice to evaluate the possible effect of TTC treatment (**Figure 2**).

**FIGURE 3:**
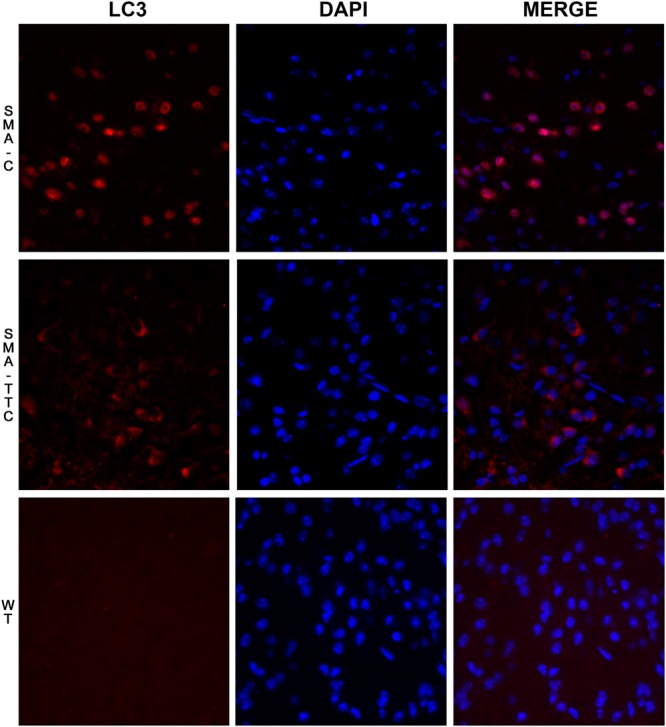
**Immunofluorescence staining for LC3 on spinal cord tissue under TTC treatment.** LC3 staining (red) was performed in WT mice and SMA mice untreated and treated with TTC in spinal cord slices. DAPI staining was also performed (blue). A merged image of the double staining is presented. A representative image presents of three independent animals for genotype and disease stages (40X).

In spinal cord tissue (**Figure 2A**), autophagy markers were significantly upregulated in untreated SMA mice, suggesting a potential autophagy activation due to the neurodegenerative progression of the disease. Under TTC treatment, the mRNA expression levels were significantly downregulated with respect to untreated SMA mice, pointing out to an improvement of autophagy markers, which tend to reach WT levels. Furthermore, inmunofluorescence against LC3 demonstrated that the over-expression of the LC3 transcripts was accompanied to an activation of the expression of LC3 protein, suggesting an activation of the autophagy process as a compensatory mechanism (**Figure 3**). On the contrary, LC3 protein expression was not observed in wild-type animals resembling what was observed in the case of the mRNA transcript levels (**Figure 3**).

The expression levels of pro-apoptotic genes *Bax* and *Casp3* were significantly upregulated with respect to WT mice suggesting an apoptosis enhancement in this tissue due to the neurodegeneration. However, TTC treatment significantly decreased the levels of both genes in relation to untreated SMA mice, favoring an amelioration of apoptosis (**Figure 2A**).

In relation to the skeletal muscle tissue (**Figure 2B**), the significant upregulated levels of *Becn1, Lc3*, and *p62*, in untreated SMA mice were indicative of an autophagy activation, as it was observed in spinal cord tissue. TTC treatment especially improved *Lc3* levels that reached the ones observed in WT mice, suggesting a significant amelioration of autophagy process. Furthermore, in SMA mice the levels of the pro-apoptotic gene *Bax* were found significantly upregulated, while *Casp3* levels showed a tendency to be upregulated. Under TTC treatment, only the *Casp3* showed a significantly downregulation with respect to untreated SMA mice, which could indicate a lack of apoptosis activation.

### Compensatory Response of TTC Treatment for Muscular Atrophy in SMA Mice

**FIGURE 4:**
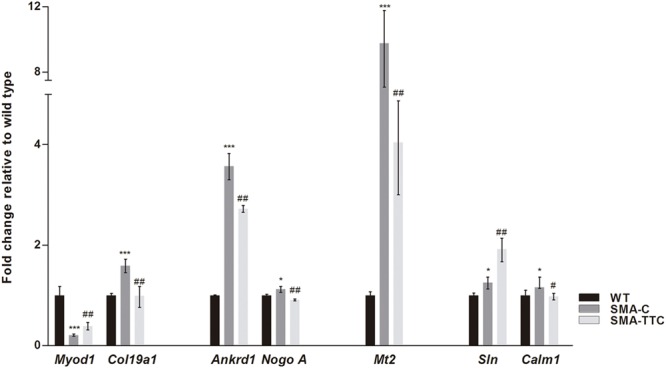
**Amelioration of genes related to muscular atrophy in SMA mice.** Relative expression values of *Myod1, Col19a1, Ankrd1, NogoA, Mt2, Sln*, and *Calm1* in WT mice (WT, black bars), untreated SMA mice (SMA-C, dark gray bars) and SMA mice treated with TTC (SMA-TTC, light gray bars) in skeletal muscle tissue. Each data point represented the mean ± SEM, *n* = 5 animals per group. WT *versus* SMA-C: ^∗^*p* < 0.05 and ^∗∗∗^*p* < 0.001. SMA-C *versus* SMA-TTC: ^#^*p* < 0.05 and ^##^*p* < 0.01.

Spinal muscular atrophy and ALS, two lethal motor neuron diseases, share affected target tissues such as the skeletal muscle. In addition, muscle weakness and atrophy have been described in SMA mouse models. Previous work from our group has described a list of genetic biomarkers for ALS disease, some of which were in close relation to muscle atrophy (Calvo et al., 2012a). Consequently, transcriptional expression levels of seven genes related to muscle atrophy (*Ankrd1, Calm1, Col19a1, Mt2, Myod1, NogoA*, and *Sln*) (Calvo et al., 2012a) were tested in skeletal muscle tissue from SMA mice (**Figure 4**).

Our results showed a significant upregulation of these genes in untreated SMA mice, except for *Myod1* that was significantly downregulated. TTC treatment reduced significantly *Ankrd1, Calm1, Col19a1, Mt2*, and *NogoA* levels, levels, and increased *Myod1* levels tending to reach WT ones, which could suggest an improvement of the muscle atrophy under TTC treatment. The significant upregulation of *Sln1* levels under TTC treatment could also indicate an improvement of the relaxation-contraction cycles, favoring an amelioration of muscle atrophy (Casas et al., 2013).

### Body Weight and Survival Rates Under TTC Treatment

**FIGURE 5:**
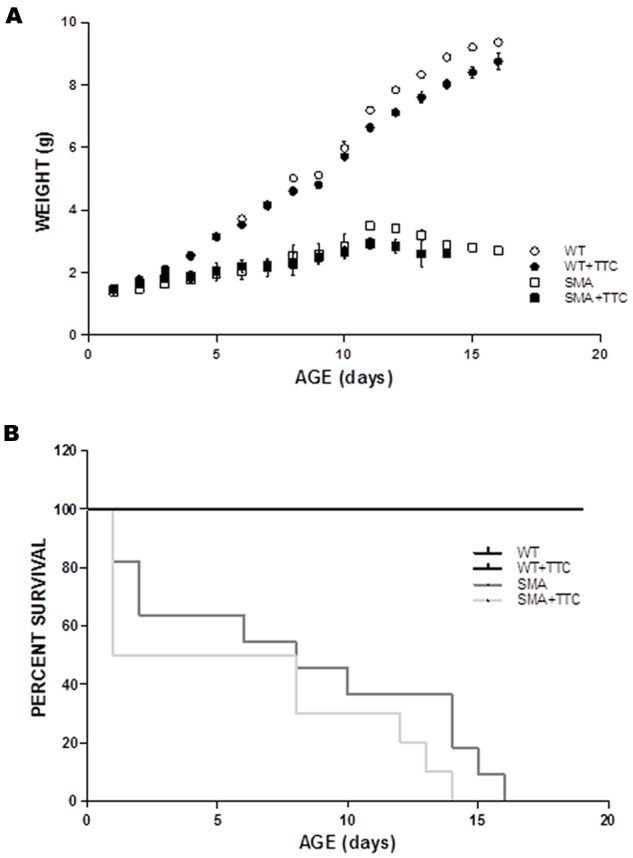
**Phenotype and survival time of treated and untreated SMA mice. (A)** Weight curves of WT (circles) and SMA mice (squares) treat (closed forms) and untreat (open forms) with TTC. All plots are shown as means of weight at each day with error bars representing standard deviation. ^∗^*p* < 0.05. **(B)** Kaplan–Meier survival analysis of WT and SMA mice with and without TTC treatment.

To evaluate the possible effect of TTC treatment in the SMA mice phenotype, the body weight was registered daily along disease progression. The results showed that the intramuscular injection of TTC-encoding plasmid at P1 did not significantly affect the body weight of WT or SMA mice during the first ten days of life (**Figure 5A**). Nevertheless, the body weight of WT mice significantly reached low levels from P12 until P16, while in treated SMA mice, a significant decrement was only detected at P11 and then a modest but no significant improvement in the body weight was observed from P12 until P16, with respect to untreated SMA mice.

Regarding the survival time, the data showed no significant differences between WT or SMA mice after TTC injection (**Figure 5B**). Albeit, it should be pointed out the high mortality rate and the variable life expectancy of the SMA pups, and therefore less severe animal model of the disease may allow a long-lasting monitoring of TTC effects.

## Discussion

In recent years the neuroprotective effects of TTC have been described in relation to the antiapoptotic and survival pathways, suggesting a similar way of action as in the case of neurotrophins in animal models of several neurodegenerative diseases (Chaib-Oukadour et al., 2004). In this sense, it has been demonstrated in neurons of mSOD1 mouse model of ALS that the neuroprotective role of TTC was possibly due to its pro-survival and anti-apoptotic properties (Moreno-Igoa et al., 2010; Herrando-Grabulosa et al., 2013). A recent work in a mouse model intermediate of SMA showed that this disease in skeletal muscle emerges before pathology in spinal cord, including loss of motor neurons. (Fayzullina and Martin, 2014). This cellular loss may be mediated by apoptosis or autophagy, therefore therapeutic strategies based on modulating these molecular mechanisms may have potential beneficial effects in the SMA pathology or disease progression. Moreover, the increment of SMN observed after non-viral gene therapy treatment with pCMV-TTC could be improved the beneficial effects related to TTC.

Autophagy has come to the forefront in motor neuron diseases as a common molecular pathway altered in degenerating motor neurons. The loss of critical genes involved in the execution of the autophagy pathway in the central nervous system results in profound severe neurodegenerative diseases (Komatsu et al., 2006). Moreover, induction of autophagy has been reported in numerous models of neurodegenerative diseases, and may be a component of the cellular response to depleted SMN levels (Custer and Androphy, 2014). At P7, the SMA pups were still active and significantly smaller than their phenotypically normal siblings (2.17 ± 0.28 vs. 4.18 ± 0.11), but signs of muscle weakness were observed compared to their normal littermates. Moreover, their relative mobility insured that they were still gaining access to milk. The presence of milk in the stomach was also visible through the skin, preventing any potential autophagy induction due to poor nursing following the onset of major motor coordination losses. In a severe model of SMA, *in vitro* experiments showed a deregulated autophagy in spinal cord motor neurons (Garcera et al., 2013). Moreover, LC3-II and p62 protein levels were increased in lysates of spinal cord from a severe mouse model of SMA indicating that autophagy is dysregulated (Custer and Androphy, 2014). In accordance with these results, our data revealed a significant increase in the expression levels of all autophagy markers in spinal cord and skeletal muscle tissues and therefore an activation of this process. Although autophagy remained activated under TTC treatment in SMA mice, the gene expression profile observed in treated SMA mice tended to reach WT levels. In case of muscle, TTC treatment decreased *Lc3* expression to WT ones. This reduction of *Lc3* levels suggested that TTC was able to reduce the pathological autophagy until a constitutive autophagy, since Lc3 is considered the main marker of autophagosomes.

In relation to apoptosis, the anti-apoptotic properties of TTC were evidenced by *in vitro* studies in cultured neurons in which TTC preserve mitochondrial function decreased nuclear fragmentation and reduced activation of pro-Casp3 (Chaib-Oukadour et al., 2004). Similarly, *in vivo* experiments in a model of Parkinson’s (Mendieta et al., 2009) or ALS’s (Moreno-Igoa et al., 2010) diseases, showed an anti-apoptotic effect of TTC. In regard to apoptosis and neurodegenerative disorders, a deregulation of this process is associated with a long list of pathologies (Marino et al., 2014). In SMA disease, genetic studies in mice (Kerr et al., 2000; Anderton et al., 2013) support a role for programmed neuronal death. Thus, in the central nervous system of SMA mouse models, elevated levels of pro-apoptotic genes and an enhancement of apoptosis have been observed (Tsai et al., 2006). Moreover, a significant loss of large motor neurons was observed in the spinal cord from SMNdelta7 mouse at P7 (Baumer et al., 2009), and 2 days later this loss was over 50% (Edens et al., 2014). Furthermore, in the skeletal muscle of severe SMA model mice, the presence of apoptotic cell death signs was detected (Dachs et al., 2011). In connection with these results, we observed that the pro-apoptotic gen *Bax* was upregulated as well as the gen *Casp3*. Both genes encode for the “executioner” proteins in apoptosis, suggesting that apoptosis was activated. In relation to the skeletal muscle, a recent work using the “Taiwanese” SMA mouse model, detected at birth DNA damage that was getting worse until P6 when the muscle exhibited cell death (Fayzullina and Martin, 2014). However, in our SMA mice, the upregulation of *Bax* together with the lack of upregulation of *Casp3* suggested an absence of apoptotic cell death in untreated SMA mice, which is in accordance with the results obtained by Hayhurst and co-workers that revealed normal proportion of apoptotic cells. After TTC treatment, the expression levels of *Bax* and *Casp3* in spinal cord and *Casp3* in skeletal muscle were downregulated untreated SMA mice, suggesting that TTC favored the normalization of the expression levels of both genes and therefore played a relevant role in the modulation of apoptosis in this animal model.

Although the importance of motor neuron pathology is well-established in SMA, a cumulative body of work supports the involvement of other cell types, including myocytes (Hamilton and Gillingwater, 2013; Martinez-Hernandez et al., 2013; Iascone et al., 2015). In SMA disease, muscle weakness and atrophy are also principal pathological hallmarks. In this way, in mouse models of the disease, several works reported an abnormal skeletal muscle development (Boyer et al., 2013).

Consequently, our next step was the evaluation of the expression of several genetic biomarkers related to muscular atrophy, previously characterized by our group (Calvo et al., 2012a). TTC administration significantly reduced the transcriptional level of *Myod1*, an early marker of myogenesis (Manzano et al., 2011), and *Col19a1* which increasing levels are related to the regenerative response to muscle damage (Sumiyoshi et al., 2001), shifting their expression toward the wild-type levels. Additionally, after TTC treatment the expression levels of *Ankrd1*, a marker of muscle damage (Laure et al., 2009), and *Nogo A* that accelerates the progressive failure of motor neuron innervation (Pradat et al., 2007) decreased to reach WT ones, suggesting that TTC could ameliorate muscle atrophy. Finally, TTC treatment modified the expression of *Mt2, Calm1* and *Sln* genes which are also associated to muscle atrophy (Hyldahl et al., 2010; Casas et al., 2013).

In summary, non-viral gene therapy based on TTC improved the expression levels of main genes related to autophagy and apoptosis in SMA mice. In particular, in response to the neurodegenerative progression in SMA mice, TTC treatment modifies the expression of autophagy and apoptotic genes. Additionally, TTC reduced the expression of autophagy markers and pro-apoptotic genes in spinal cord while in skeletal muscle TTC was able to downregulate the expression of the main marker of autophagy (*Lc3*) to WT levels, as well as the expression of the apoptosis effector, *Casp3.* Furthermore, in the skeletal muscle tissue of treated SMA mice, TTC showed a compensatory effect in the expression of genes involved in muscle damage response, oxidative stress and calcium homeostasis. These preliminary findings provide new insights into the effect of TTC in the spinal cord and the skeletal muscle tissues in SMA disease and suggest the need for further experiments to accurate study the effect of TTC in this disorder.

## Author Contributions

Conceived and designed the experiments: SO, ACC, RO, and JA. Performed the experiments: SO, AR, MH-G, and RM. Analyzed data: SO, ACC, and AR. Contributed reagents/materials/analysis tools: JA, PZ, and RO. Wrote the manuscript: SO, ACC, EFT, and RO.

## Conflict of Interest Statement

The authors declare that the research was conducted in the absence of any commercial or financial relationships that could be construed as a potential conflict of interest.

## Abbreviations

SEM: standard error of the mean
SMA: spinal muscular atrophy
SMN: survival motor neuron
TTC: *>C*-terminal fragment of the tetanus toxin
WT: wild type
