# Supplementary material for: Neuroprotective Effect of Non-viral Gene Therapy Treatment Based on Tetanus Toxin C-fragment in a Severe Mouse Model of Spinal Muscular Atrophy
Source: Front Mol Neurosci. 2016 Aug 24;9:76. doi: 10.3389/fnmol.2016.00076 (PMC4995219; doi:10.3389/fnmol.2016.00076)

Supplementary Material

S1. Effect of TTC-encoding plasmid in SMN expression.

To analyze the effect of TTC-encoding plasmid in SMN gene expression, pCMV-TTC plasmid were injected in a mouse model of motor neuron disease (B6SJLTg(SOD1*G93A)1Gur/J). At 60 days of life, mice were injected intramuscularly with 100 µg of naked DNA enconding for pCMV-TTC or non-coding pCMV plasmids into the quadriceps femoris muscles (one injection with 50 µg per muscle). Ten days later (70 days of life), mice were euthanized and skeletal muscle and spinal cord tissues were dissected for RNA extraction. The expression of SMN gene in skeletal muscle and spinal cord tissues was assessed by real-time PCR as above described.

The results obtained showed that 10 days after non-viral gene therapy with pCMV-TTC, the expression of SMN gene was significantly increased in muscle (*$p<0.05$) and spinal cord (***$p>0.001$) respect to wild type mice.

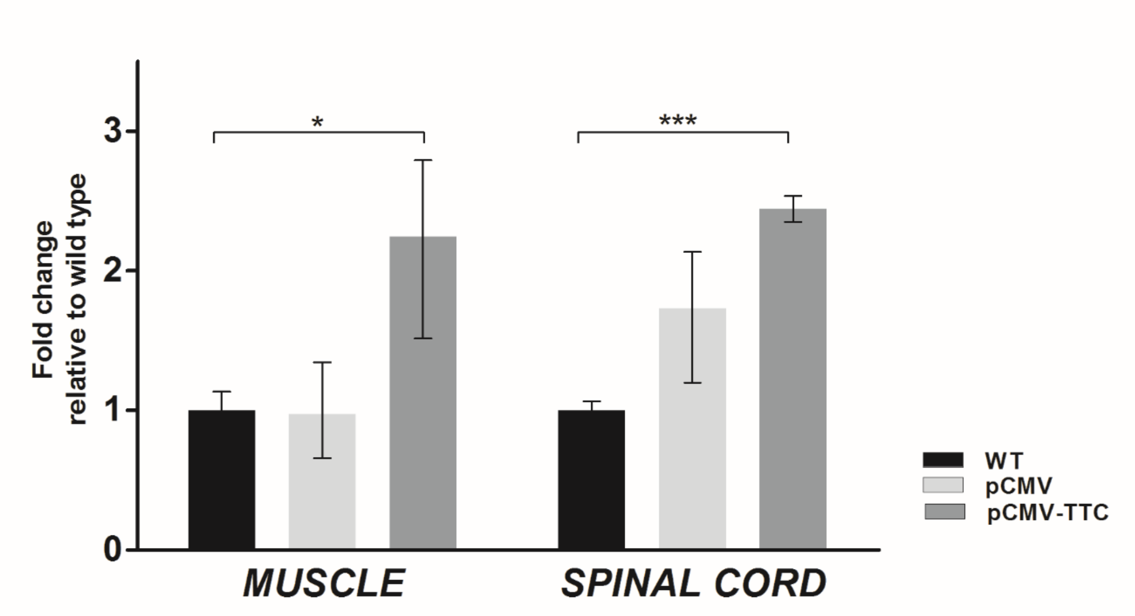

Supplement: Supplementary file 1 [file Image_1.PDF]
